# Establishing a 130-Bed Field Intensive Care Unit to Prepare for COVID-19 in 7 Days in Bahrain Military Hospital

**DOI:** 10.1017/dmp.2020.297

**Published:** 2020-08-12

**Authors:** Nayef A. Louri, Jalal Abdulla Alkhan, Hayyan Hamad Isa, Yaser Asad, Abdulla Alsharooqi, Khalifa Ahmed Alomari, Nahed Kamal Hasan, Fahad Bin Khalifa Al Khalifa, Ghaida Fareed Ahmed, Muna Yaqoob Alasmi, Dana Khalifa Al-Khalifa, Khalid Bin Ali Al Khalifa

**Affiliations:** Bahrain Defence Force Royal Medical Services, Military Hospital, Bahrain

**Keywords:** Bahrain, COVID-19, field-ICU, isolation facility

## Abstract

This article reports the establishment of an isolated, fully functional field intensive care unit (FICU) unit equipped with all necessary critical care facilities as a part of the national pre-emptive preparedness to treat an unexpected surge outbreak of coronavirus disease 2019 (COVID-19) patients in Bahrain. One floor of an existing car parking structure was converted into a 130-bed FICU set-up by the in-house project implementation team comprised of multidisciplinary departments. The setting was a military hospital in the Kingdom of Bahrain, and the car park was on the hospital premises. The FICU contained a 112-bed fully equipped ICU and an 18-bed step-down ICU, and was built in 7 d to cater to the intensive care of COVID-19 patients in Bahrain.

The first suspected case of novel coronavirus disease 2019 (COVID-19) was reported by China to the World Health Organization (WHO) on December 31, 2019.^1,2^ Thereafter on January 30, 2020, the WHO declared the outbreak a Public Health Emergency of International Concern. By March 7, 2020, approximately 100 countries reported COVID-19 cases, and the total global COVID-19-positive cases crossed 100,000. On March 11, 2020, the WHO declared the disease as a pandemic owing to its rapid spread in the global scenario.^3^ Since then, COVID-19 has spread to 215 countries around the globe with a rapidly increasing number of daily confirmed cases. Bahrain recorded its first COVID-positive case on February 24, 2020^3^ and by the end of March 31, 2020, the number of positive cases increased to 449.^3^ Bahrain is 1 of the few countries that initiated extensive contact tracing of all positive cases and kept them in isolated quarantine facilities on a very early stage of disease spread. In addition, a Bahrain National Task force (BNTF) was set up to monitor the progress, containment, and treatment of COVID-19.

Per the WHO patient data for COVID-19, approximately 15% of COVID-positive patients would require intensive care unit (ICU) care.^4^ Going by this estimate and the rate of increase of positive patients in Bahrain, it was estimated that the existing ICU facilities would not be sufficient during the outbreak. As of April 1, 2020, ICU facilities available in 4 government hospitals in Bahrain sum up to 161 ventilator-equipped beds (VEB) with an estimated 60% occupancy rate for normal medical intensive care requirement. Thus, there was an immediate requirement of additional ICU beds as part of the Bahrain National pre-emptive preparedness plan to match up to the WHO reported COVID-19 patient treatment data. In March 2020, BNTF took up the initiative to establish 4 field intensive care units (FICU) with additional 500 ICU beds for the outbreak. The Bahrain Defence Force Royal Medical Services (BDF-RMS) military hospital was given the responsibility to establish a fully equipped FICU in its premise as a part of the national plan to combat the outbreak. Henceforth, a project design and implementation committee was formed in the BDF-RMS on April 1, 2020, to implement the project in the shortest possible time. This study will discuss the step-by-step approach of setting up the FICU facility with the available resources within 7 d. The objective of this study is to enable the replication of this model of emergency infrastructure preparedness plan to treat COVID-19 patients.

## METHODS

### Hospital Location

The BDF-RMS has 3 arms: (1) military hospital, (2) cardiac center, (3) Field Medical Battalion. The military hospital provides tertiary health care to the Bahrain defence forces and civilian population and has 24 VEBs with 60-70% occupancy. The challenge of providing COVID-19 ICU facility in a military hospital was 2-fold. (1) Insufficient VEBs in case of outbreak. (2) Risk of nosocomial infection and catastrophic shutdown of medical services if COVID-positive patients are admitted along with other patients in the existing ICU. The WHO manual to set up and manage a severe acute respiratory illness (SARI) treatment center and SARI screening facility in health-care facilities requires an isolated closed space within the hospital that can be upgraded temporarily to a FICU.^5,6^

### Setting up of Project Planning and Construction Committee: Day 1

A FICU project planning and implementation committee was set up under the leadership of the director of BDF-RMS and headed by Colonel Nayef Louri. The other committee members consisted of the Administration of BDF-RMS and heads of different medical and nonmedical departments, including ICU, Finance, Projects and Planning, Biomedical, IT, Mechanical, Electrical, and Nursing.

### Location Criteria as per WHO Guidelines

The criteria were as follows: ensure that the site is as close as possible to the main entrance of the health facility to centralize all entrances; ensure easy access for patients, visitors, and staff with guaranteed security; aim for a unidirectional flow for all patients and visitors accessing the health facility; and the location should ideally be inside the premises of the health-care facility for optimum manpower management but should ideally be an isolated structure separated from existing patient treatment areas to prevent possible cross-infection.

Considering all the above criteria, different locations inside the premise of the BDF-RMS, including the existing patient wards were considered, but none of them were deemed fit. The urgency of the situation prevented the construction of a new separate structure in quick time. Hence, a noble idea was conceived to consider converting 1 floor of the existing multilevel car park building present inside the hospital premises to an FICU. The precast floor and roof had enough structural load bearing capacity for medical equipment and human resource use in FICU unit. The FICU unit was allotted to the 3rd floor of the car park with staff amenities on the 4th floor.

### Architecture and Design

The FICU was constructed on the 3rd floor of the car park building, which has a total of 4 floors. The total surface area covered was 3260 m^2^. The overall capacity of the FICU was 130 beds divided into 2 blocks. Block A for ICU patients contained 112 VEB for patients in need of intensive care. Block B for recovering patients contained 18 beds as a step-down ICU for nonventilated critically ill COVID-19–positive patients. The step-down unit was completely separated from the ICU section by transparent glass panels and accessed by sensor-activated controlled access doors. Entry was maintained through the FICU with separate exit and patient amenities. The FICU area was designed with free-standing air conditioning and exhaust ventilation to maintain negative pressure and reduce airborne viral load with continuous filtration. This also ensured adequate air change to maintain air quality inside the FICU. Environmental health specialist and infection control specialist of the hospital were consulted to recommend the guidelines for airflow, sluice, water, and overall sanitization of the FICU. The beds were the motorized type for patient positioning and were equipped with a piped medical oxygen supply, patient monitor, ventilator, suction machine, DVT machine, and patient trolley.

**FIGURE 1:**
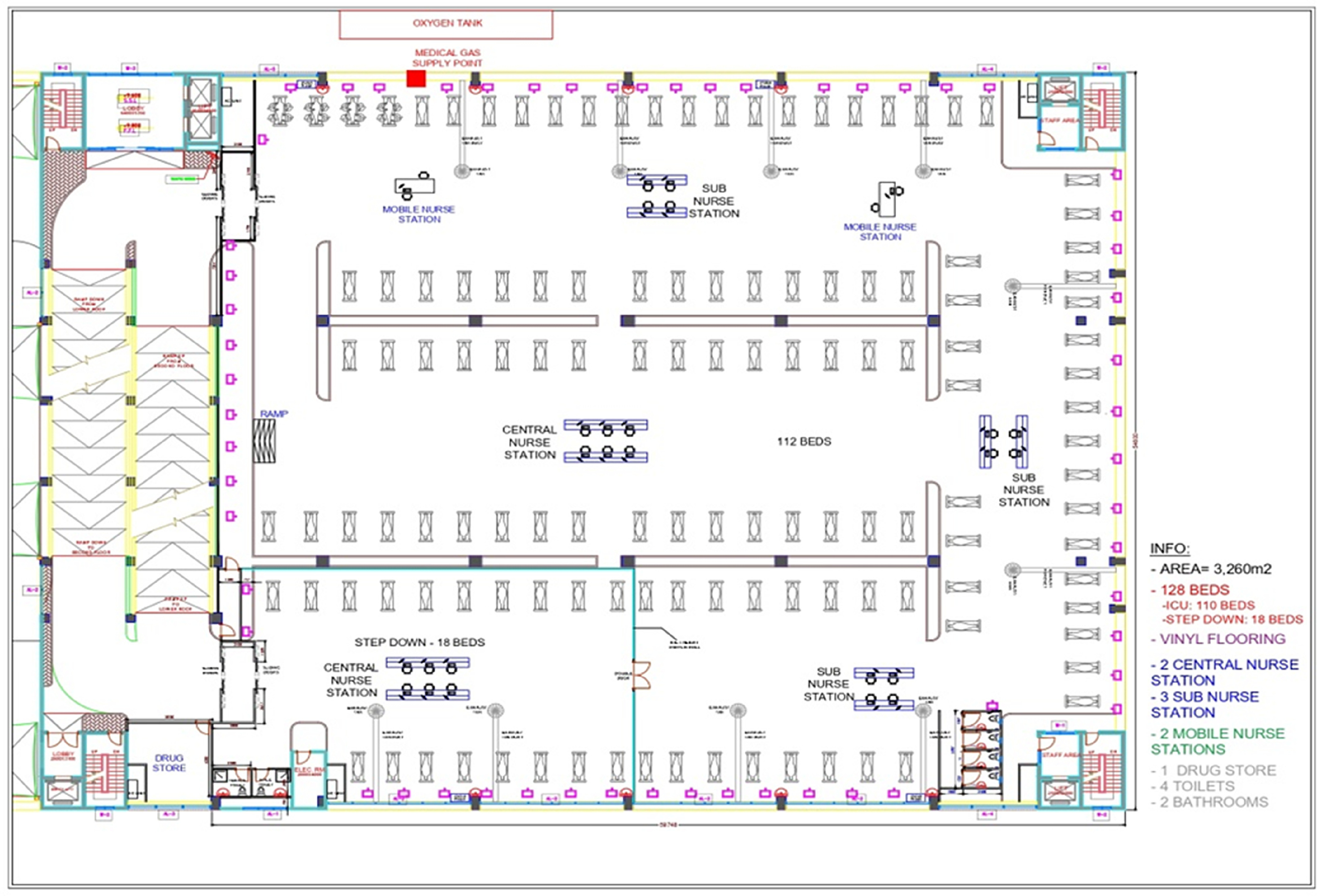
Complete Layout Plan of FICU.

Other required medical equipment and facilities in the FICU included 4 CRRT machines, 2 portable ultrasonography machines, 1 ECMO machine, and 1 portable X-ray machine. The FICU also contained 8 nursing stations equipped with desktop computers, platform mobile computers, printers, label printers, public announcement system, and intercom telephones. The computerized patient data management system was connected to the hospital main server. A dedicated drug store was also set up in the FICU. Two dedicated ambulances were procured for transporting only COVID-19–positive patients to and from the facility. Staff amenities, such as male and female changing rooms, shower rooms, and staff lounge, were located on the rooftop of the FICU. These were interconnected to the FICU through 2 different elevators and adjacent staircases. Sixteen portable hand-washing basins were installed inside the FICU for ease of hand-washing for staff. The complete layout plan of the FICU is given in Figure 1.

### Management of Infection Control Measures and Safety of Health-Care Providers

The safety of patients as well as health-care providers was given utmost priority in designing the FICU. The hospital infection control specialist team was involved during the design stage to recommend infection control measures to be implemented in the FICU. The infection control team also was assigned responsibility for continuous monitoring of environmental cleaning and sanitization of FICU during operation as per the COVID-19 infection control guidelines. In addition, the following measures were taken to minimize the chances of cross-infection from patients to health-care workers.

#### Separate Unidirectional Entry and Exit for Patients

All the patients were transported to FICU through dedicated ambulance only. The ambulance used the ramp of the existing car park to directly reach the patient entry point of the FICU that has double door access to prevent entry of outside air directly into the FICU. At the entry point, the patients were shifted from the ambulance trolley to FICU trolley and thereafter to FICU bed. The ambulance, along with paramedics in it, was then taken to the rooftop of the FICU for complete disinfection, and then the ambulance was allowed to exit through another ramp to the ground floor.

#### Separate Unidirectional Entry and Exit of On-Duty Staff

**FIGURE 2:**
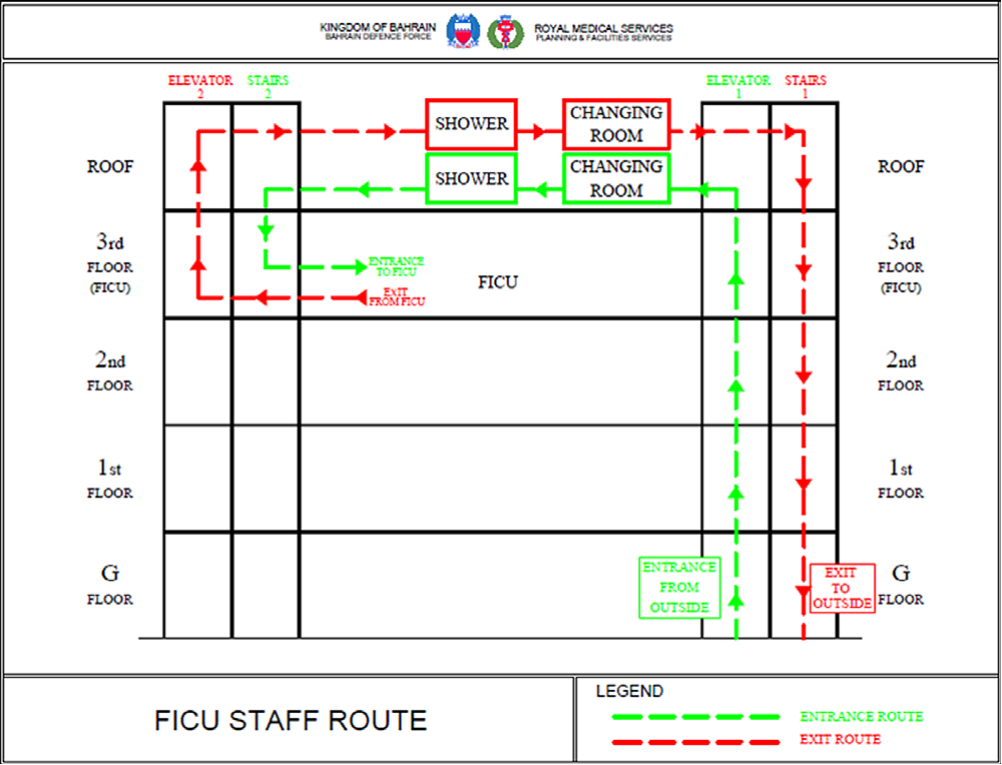
Diagrammatic Description of Unidirectional Movement of Duty Staff.

The pathway for the entry and exit of duty staff was unidirectional and used 2 different elevators and adjacent staircase units present at the opposing ends of the FICU building. As a rule, elevators were always used for moving upward and staircases for moving downward, to maintain unidirectional movement. Elevator 1 was assigned as the clean elevator. This was used only by FICU duty staff to enter the FICU building at the beginning of their duty shift. The on-duty staff get their thermal screening done just before entering elevator 1 at the ground floor. Then they move up in elevator 1 directly to the rooftop of the FICU. The rooftop contained 3 porta cabins designated for changing room, shower room, and staff lounge separately for male and female staff. After reaching the rooftop, the staff members first need to enter the change room, store their clothes in individual lockers, change to scrubs, and don complete personal protective equipment (PPE). Then they move to the opposite side of the rooftop and walk down a separate staircase (staircase 2) to enter the FICU situated on the 3rd floor just below the rooftop level. After their duty shift, the staff are instructed to enter the after-duty change-over area of the FICU adjacent to elevator 2. Here, the staff wearing PPE enter a disinfectant spray area, they retain their N95 mask, discard their PPE in the designated place, and take elevator 2 to the rooftop. Staff are required to enter the changing room and collect their clothes, and then enter the shower room to shower, change to normal clothing, and directly exit from rooftop to ground floor of the building using staircase 1. Staff members requiring a washroom facility during duty hours can use the washrooms located on the rooftop while wearing PPE. All the staff working in the FICU were tested for COVID-19 once a week. The unidirectional movement of staff is described in Figure 2.

#### Provision of Pass-Box for Pickup and Drop of all Materials

A double-door pass-box was made in the wall near the main entrance of the FICU to transfer-in and transfer-out all materials, including patient swabs and blood samples to the laboratory for investigation. This was done to prevent unnecessary movement of personnel in and out of the unit and prevent unnecessary opening of the main doors. This also helped in maintaining the negative pressure inside the FICU.

#### Biomedical Waste Disposal

The provision of disposal of biomedical waste was done as per international guidelines. The wash basin water outlet pipes were cored through the floor and connected to the hospital sewage system. All the biodegradable biomedical wastes (gauze, body fluids, etc.) were collected in color-coded biomedical waste bags kept in disposable bins and were disposed of with the help of a biomedical waste disposal system called Cycloflush^TM^. A separate pathway was made for the transfer of clean linen and dirty linen into and out of FICU. A separate elevator (elevator 3) was used for transport of dirty linen to laundry and CSSD. Almost all items used in FICU were disposable unless no disposable was available for that item.

### Management of Patient Data Recording and Storage by IT linkage

IT networking was done with the nearest network switch. Up-linking was done with fiber optics, and the network switch was installed. Network cabling was done to the nursing station and Wi-Fi access points inside the FICU. Eight nursing stations were equipped with 36 desktops personal computers (PCs) and 4 mobile PCs for patient record management. The PCs were connected to the hospital patient record management software for remote access. Provision was made to record all patients’ data, treatment notes, investigations, and medication prescriptions digitally through the hospital patient management software. The existing air-conditioned room for the electrical system of the car park was used to install the network switch. Separate access control and staff attendance systems were also installed at the FICU to enable duty staff marking their biometric attendance and not to move to other departments, thus preventing cross-infection in the hospital premises. The entire project was divided into 14 sub-projects as listed in Table 1. The responsibility of completion of sub-project was delegated among project implementation team members. The head of the project coordinated with each sub-project team leader for streamlining of work completion.

**TABLE 1:**
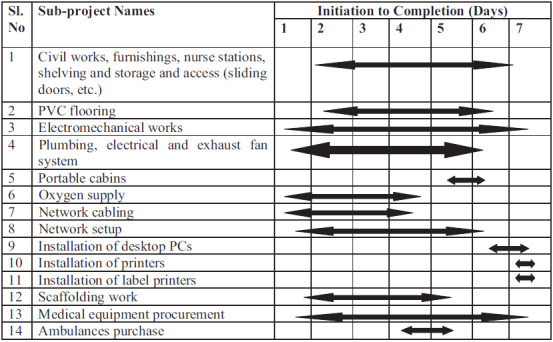
Project Implementation Timeline Explained in Gantt Chart

### Management and Training of Human Resources

In 2019, the hospital had established a large state-of-the-art medical training center to provide professional training and certify the skills of medical professionals using the latest simulation-based training modules and refresher courses. After the outbreak of COVID-19, this center was fully used to train a large number of in-house and other hospital medical staff on COVID-19 to prepare a large pool of trained staff to treat COVID-19 patients during pandemic in the country. The trainers designed 6 COVID-19–specific training courses for nurses and doctors: (1) basic knowledge of ICU care for non-ICU physicians, (2) handling patients on mechanical ventilators, (3) respiratory care course, (4) total parenteral nutrition orientation training, (5) respiratory care of patients on mechanical ventilators, (6) course of donning and doffing of PPE for cleaners, patient attendants, central sterilization, catering, and ambulance staff.

The training schedule was prepared in a continuous staggered manner to enable all the staff to attend the courses in addition to their normal duties. The courses were given as per the duty requirement of each staff. The 2-d course on basic knowledge of ICU was attended by all the non-ICU physicians. In addition, multiple sessions of mock drills were conducted for all the nurses in the FICU to get accustomed to the working conditions and entry-exit procedure in the new FICU. The training sessions were conducted by a team of experienced simulation training instructors, including intensivists, nursing instructors, and allied health-care trainers of BDF military hospital medical training center. The health-care providers from 3 other allied hospitals of Bahrain were also trained in the training center to prepare adequately trained manpower to deal with surge capacity. The details of the training programs are given in Table 2.

**TABLE 2:**
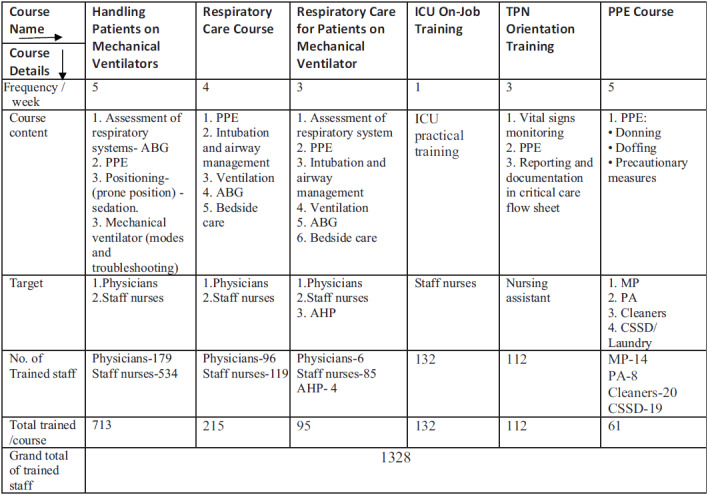
Details of COVID-19–Specific Training Program

Abbreviations: ABG, arterial blood gas; AHP, allied health-care provider; CSSD, Central sterile Supplies Department; ICU, intensive care unit; MP, military police; PA, patient attendant; PPE, personal protective equipment; TPN, trained practical nurse.

### Creation of Health-Care Provider Pool for COVID-19 Patient Management and Staff Escalation Plan

The total manpower of the hospital was divided into 2 categories: COVID-19 core team and COVID-19 back-up team. Many doctors, nurses, allied health-care personnel, including respiratory therapists, physiotherapists, infection control nurses, dieticians, and X-ray technicians, were enrolled into the COVID-19 team. A total of 610 trained nurses and 167 physicians were enrolled to form the COVID-19 treatment team. The physician team included doctors from different specialties, including intensivists, anesthesiologist, medicine specialist, emergency, general practitioner, and surgery. The COVID-19 back-up team was also given COVID-19 training and kept as standby to supplement or replace the COVID-19 core team members if required. A staff escalation plan was prepared to pull out the required number of staff from normal duty and deploy in the FICU as per the requirement. The patient escalation plan was divided into 3 phases, depending on the number of patients admitted at any given point: (1) yellow phase for admission of 1-30 of patients; (2) orange phase for 31-70 patients; and (3) red phase for 71-130 patients. This was done to manage the staff allotment as per the surge of patients admitted to the FICU. The nurses and physician escalation plans are given in Table 3 and Table 4, respectively.

**TABLE 3:** Nurses Allotment Escalation Plan for FICU: Assignment at Each 12-Hour Shift

Phase Yellow	Total No. of Patients in FICU	No. of Additional Nurses to Be Called	Total Nurses to Be on Duty	Nurse-to-Patient Ratio
Yellow 1.1	1-10	10	10	1:1
Yellow 1.2	11-15	5	15	
Yellow 1.3	16-20	5	20	
Yellow 1.4	21-30	10	30	
**Phase Orange**				
Orange 2.1	31-40	10	40	1:1
Orange 2.2	41-50	10	50	
Orange 2.3	51-60	10	60	
Orange 2.4	61-70	10	70	
**Phase Red**				
Red 3.1	71-90	20	90	1:1
Red 3.2	91-110	20	110	
Red 3.3	111-130	20	130	

**TABLE 4:** Physicians’ Allotment Escalation Plan to Handle FICU Surge Capacity

	Pandemic Phase: 1^st^ Phase FICU Capacity (<30 Beds or 25% Capacity)			
Phase Yellow	Total No. of Patients in FICU	ICU Intensivists	Other Physician	Total
Yellow 1.1	1-10	5	5	10
Yellow 1.2	11-15	6	6	12
Yellow 1.3	16-20	7	7	14
Yellow 1.4	21-30	8	8	16
**Phase Orange**	**Pandemic Phase: 2** ^**nd**^ **Phase FICU Capacity (<70 Beds or 55% Capacity)**			
Orange 2.1	31-40	9	9	18
Orange 2.2	41-50	10	10	20
Orange 2.3	51-60	11	11	22
Orange 2.4	61-70	12	12	24
**Phase**	**Pandemic Phase: 3** ^**rd**^ **Phase FICU Capacity (130 Beds or 100% Capacity)**			
Red 3.1	71-90	13+	20	33
Red 3.2	91-110	13+	30	43
Red 3.3	111-130	13+	40	53
**Manpower Deployment in Each Shift (12-Hour Shift) for Full Capacity FICU**				
**Patients**		**ICU/Anesthesia Physician and Other Physician**	**Nurses**	**Respiratory Therapist**
For 130 (ventilated patients)		27	210	12

### Updated Statistics on COVID-19 Infected Patient Data

As of June 21, 2020, Bahrain has reported 21,764 positive cases of COVID-19 infection.^7^ Of the total positive cases, 16,416 patients have been treated and discharged, and the number of active cases is 5281. The total number of deaths reported is 64. Of the 5281 active cases, 5249 cases are classified as stable, and the other 32 are classified as critical. Of the 5249 stable patients, 169 patients are receiving active treatment, and the other 5080 patients are in isolation either in dedicated quarantine centers or in home isolation as per the updated policy of BNTF. The FICU has treated a total of 255 COVID-19 patients requiring ICU care to date. Of the 255 treated patients, 98 patients were shifted to other facilities after their condition improved, 47 patients were discharged home, and 18 deaths were reported. Currently, there are 91 in-patients in the FICU undergoing treatment.

## RESULTS

**FIGURE 3:**
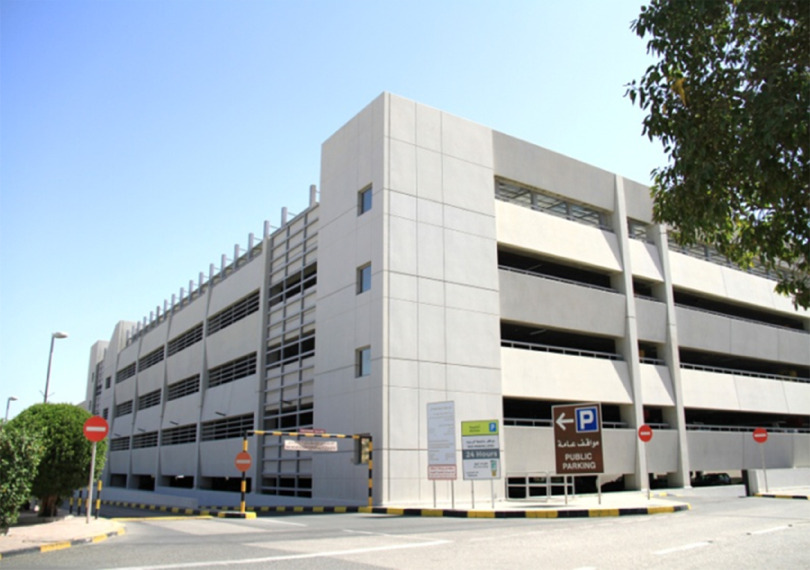
Car Park Before Construction of FICU.

**FIGURE 4:**
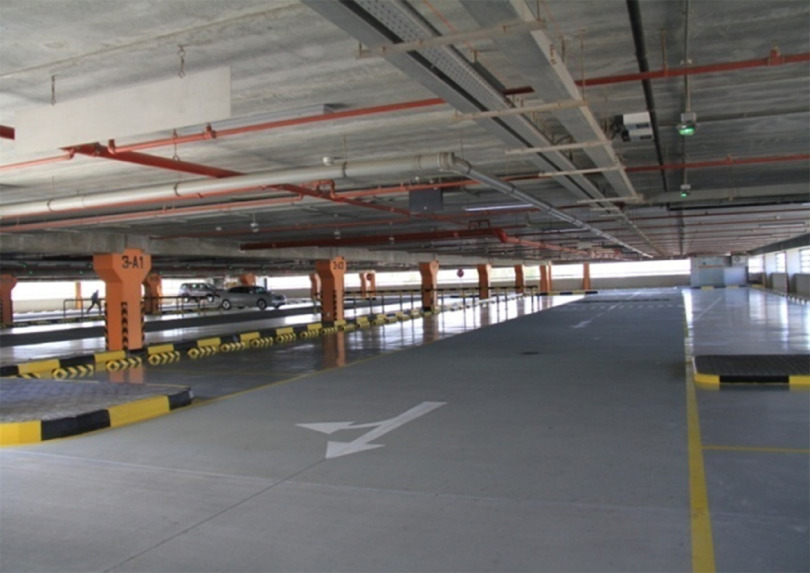
Inside View of the Car Park.

**FIGURE 5:**
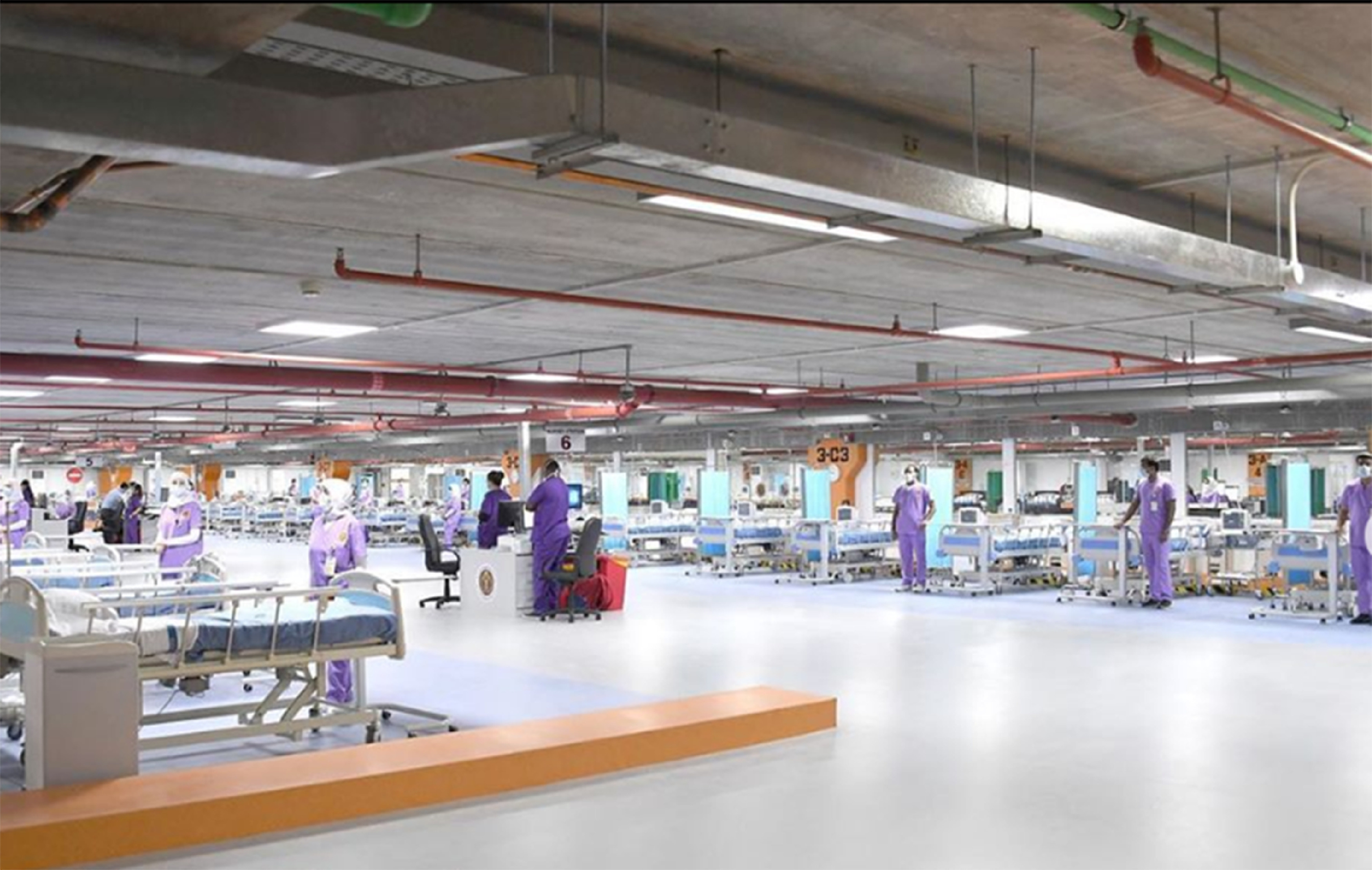
Fully Functional FICU.

The whole project was completed in 7 days. Construction and assembly commenced on April 1, 2020, and the project was completed on April 8, 2020, and officially inaugurated on April 9, 2020. The photograph of FICU before and after construction is shown in Figures 3-5.

## CHALLENGES

The implementation team faced the following challenges and managed to overcome those with innovative approaches. These points should be kept in mind while implementing such types of projects in future.

- Logistics for getting the nearest source of electricity for project requirement.
- Height of ceiling. As it was a car park, it had relatively low height ceiling. Therefore, we had to customize our air-conditioning and exhaust ventilation according to height of ceiling.
- Coring of cast on the floor and ceiling for installation of different services was a big challenge.
- Managing the escalation and de-escalation plan of staff as per the number of patients admitted in FICU was a major challenge. A separate nursing administration hierarchy was created for FICU with a team leader followed by 2 shift supervisors and shift managers followed by nursing staff.
- Managing staff de-escalation plan and replacement. Ensuring no staff getting infected during duty was a major challenge for the administration. Any staff being infected and becoming an asymptomatic carrier can result in major disruptions in service and require immediate staff replacement that may not be feasible. Hence, a comprehensive staff monitoring system was put in place. It had 2 components: daily online reporting of COVID-19 symptoms and physical monitoring at the entry point of the FICU. An online COVID-19 monitoring system was created by the IT department wherein all staff members were required to log in and report if he/she has any COVID-19 symptoms. They were also required to inform their respective supervisor by phone. Then, an immediate COVID-19 test was done, and the staff was kept in self-quarantine until the test results were obtained. In addition, a physical monitoring desk was set up near the entry point of the FICU to prevent any staff with suspected COVID symptoms entering the FICU. A pool of back-up support staff was also trained and kept ready to replace any member of the FICU COVID core team.

## DISCUSSION

It was a learning experience for the project implementation team to convert the car park into a FICU unit in such a short period of time, as no previous template for such a type of project was present in the published literature.^8^ The WHO practical manual to set up and manage a SARI treatment center and SARI screening facility in health-care facilities served as the guiding document for project design. Successful establishment and functioning of the FICU involved 3 main aspects: (1) location; (2) feasibility of connecting essential services, such as electricity, water, biomedical, waste management, etc.; and (3) training of manpower.

### Ventilation

The location of the multilevel car parking as a separate isolated structure inside the hospital campus near to the entrance, with separate entrance and exit from the existing treatment area fulfilled the ideal location as per WHO practical manual. The separate air conditioning system and separate exhaust ventilation helped in minimizing the chance of cross-infection to other patients being treated inside the hospital. In addition, the car park was surrounded by open space from all sides, facilitating adequate ventilation. Previous studies also proved the direct correlation between better air distribution and exhaust ventilation to reduced rate of cross-infection and more safety to the health-care providers.^9,10^ This important aspect should be kept in mind while selecting the location of a temporary ICU inside the existing hospital.

### Accessibility

The second important aspect of location selection is to ensure easy accessibility for the movement of patients and medical supplies. In this regard, the FICU was well connected with wide roads to facilitate movements. The existing ramp of the car park enabled the ambulance to reach the patient entry point of FICU on the 3rd floor, and the patients were shifted directly from the ambulance to ICU beds. This system prevented an intermediate shifting of transfer-trolley near the ground floor and then being wheeled into the ICU, thereby minimizing the exposure of the health-care workers to COVID-19 patients.

### Use of Car Park Facilities (Load, Cabling, Electrical Lift, Ramps)

The structural load capacity of the car park is normally lower than other structures, such as multi-story buildings, because of the lower weight to footprint ratio of cars as compared with humans.^11^ Hence, this aspect should be kept in mind while converting a single story car park to ICU, which can have more loads with heavy equipment and patients. In our case, the original building structure was designed for multilevel car park, so it had adequate load bearing capacity of precast materials used for the floor as well as roof of the car park. The next aspect of the planning involved securing 24-h uninterrupted electricity supply to fulfill the electricity demand of the FICU. It was connected to the nearest electricity distribution point (DP) by secured tunnel cabling. Safe biomedical waste disposal is another most important point to be considered while designing the facility. The existing 2 big elevators and adjacent staircases were used to design the unidirectional movement of duty staff. This ensured the entering pathway to the FICU remained sterile, minimizing the chances of spreading infection.

### Training of Staff

The creation of an adequate number of trained staff is as important as the facility. A comprehensive plan for staff training should be a part of the whole project. It is also essential to upgrade skills and acclimatize staff to the assigned ventilators and critical care equipment and protocols. This will help in minimizing human error while treating the patients.

### Sustainability and Reallocation

Setting up a temporary FICUs like our project gives the flexibility to dismantle the fittings and use the structure as a car park again whenever the epidemic-like situation is over and there is no longer a requirement for a huge number of ICU beds. Keeping this in mind, all the fittings installed in the ICU units were removable (portable air conditioners, wash basin, nursing station, etc.).

## CONCLUSION

The pandemic will change the design and function of hospitals in the future. Efficient use of the FICU will involve periodic escalation and de-escalation of critical care teams and equipment and reallocation to appropriate in-hospital locations. Planning and design of such a structure should also consider future use of the structure after the pandemic situation is over. The successful completion of this project of creating 130 beds in 7 d serves as a template for future replication in case of sudden escalation of disease in the country. This study may also help other medical facilities to plan and set up similar temporary ICU units by converting the suitable existing infrastructure present on their premises rather than building a new structure, bringing about efficient use of time, funds, and manpower.

## Supplementary material

For supplementary material accompanying this paper visit https://doi.org/10.1017/dmp.2020.297. click here to view supplementary material
